# The RUNX1-ETO target gene RASSF2 suppresses t(8;21) AML development and regulates Rac GTPase signaling

**DOI:** 10.1038/s41408-020-0282-9

**Published:** 2020-02-06

**Authors:** Samuel A. Stoner, Katherine Tin Heng Liu, Elizabeth T. Andrews, Mengdan Liu, Kei-Ichiro Arimoto, Ming Yan, Amanda G. Davis, Stephanie Weng, Michelle Dow, Su Xian, Russell C. DeKelver, Hannah Carter, Dong-Er Zhang

**Affiliations:** 1 Moores Cancer Center, University of California San Diego, La Jolla, CA 92093 USA; 2 Biomedical Sciences Graduate Program, University of California San Diego, La Jolla, CA 92093 USA; 3 Division of Biological Sciences, University of California San Diego, La Jolla, CA 92093 USA; 4 Division of Medical Genetics, Department of Medicine, University of California San Diego, La Jolla, CA 92093 USA; 5 Bioinformatics and Systems Biology Program, University of California San Diego, La Jolla, CA 92093 USA; 6 Department of Pathology, University of California San Diego, La Jolla, CA 92093 USA

**Keywords:** Acute myeloid leukaemia, Oncogenes

## Abstract

Large-scale chromosomal translocations are frequent oncogenic drivers in acute myeloid leukemia (AML). These translocations often occur in critical transcriptional/epigenetic regulators and contribute to malignant cell growth through alteration of normal gene expression. Despite this knowledge, the specific gene expression alterations that contribute to the development of leukemia remain incompletely understood. Here, through characterization of transcriptional regulation by the RUNX1-ETO fusion protein, we have identified Ras-association domain family member 2 (*RASSF2*) as a critical gene that is aberrantly transcriptionally repressed in t(8;21)-associated AML. Re-expression of RASSF2 specifically inhibits t(8;21) AML development in multiple models. Through biochemical and functional studies, we demonstrate RASSF2-mediated functions to be dependent on interaction with Hippo kinases, MST1 and MST2, but independent of canonical Hippo pathway signaling. Using proximity-based biotin labeling we define the RASSF2-proximal proteome in leukemia cells and reveal association with Rac GTPase-related proteins, including an interaction with the guanine nucleotide exchange factor, DOCK2. Importantly, RASSF2 knockdown impairs Rac GTPase activation, and RASSF2 expression is broadly correlated with Rac-mediated signal transduction in AML patients. Together, these data reveal a previously unappreciated mechanistic link between RASSF2, Hippo kinases, and Rac activity with potentially broad functional consequences in leukemia.

## Introduction

Hematologic cancer is dominated by mutations and large-scale chromosome abnormalities affecting transcription factors, splicing factors, and epigenetic regulators, which promote malignancy through alteration of gene expression^1–4^. These altered gene expression events effectively “re-program” signaling pathways in cancer cells to promote transformation; but also cause the acquisition of dependencies that can be therapeutically exploited to inhibit malignant cell growth and survival^5^. Despite knowledge of the recurring somatic mutations affecting transcriptional regulators across cancer, the specific gene expression changes that contribute to development of individual malignancies, such as acute myeloid leukemia (AML), remain incompletely understood.

One such example that is frequent in AML is t(8;21), which occurs between the *RUNX1* (AML1) and *RUNX1T1* (ETO) genes on chromosomes 21 and 8, respectively^6^. This recurring chromosomal translocation results in generation of the aberrant oncofusion protein, RUNX1-ETO, which is an established driver of leukemia development. RUNX1-ETO primarily functions to promote leukemogenesis through assembly of large transcriptional regulatory complexes that globally alter gene expression patterns and mediate aberrant transcriptional repression of critical hematopoietic genes^7–12^.

By performing gene expression analysis using a murine AML model driven by the t(8;21)-associated oncofusion protein, we previously identified Ras-association domain family member 2 (*Rassf2*) as a putative target gene that is downregulated in RUNX1-ETO-expressing leukemic blasts^13^. The “classical” *RASSFs* (*RASSF1-6*) are among the most frequent transcriptionally altered genes in human cancer^14^. RASSF proteins are non-enzymatic adaptors defined by the presence of both a Ras-association (RA) domain and a C-terminal Salvador-Rassf-Hippo (SARAH) domain^15^. Family member RASSF1A, in particular, functions as a ubiquitous tumor suppressor that experiences transcriptional silencing through promoter hypermethylation at high frequencies in cancer^16,17^, which contributes to tumorigenesis via several mechanisms^18,19^. Other individual RASSFs display more nuanced cell-type-specific expression patterns and functions^20,21^. RASSF2, similar to RASSF1A, is transcriptionally silenced in several cancer types and knockdown has previously been demonstrated to promote cell proliferation and more aggressive phenotypes in lung cancer^22^. Although linked with both oncogenic Ras signaling^23^ and the Hippo tumor suppressor pathway^24,25^, the molecular functions of RASSF2 remain poorly understood and have not been investigated in leukemia. Here, we define *RASSF2* as a transcriptionally repressed target gene of the RUNX1-ETO fusion protein and demonstrate the unique functional importance of this event to the pathogenesis of t(8;21) AML. Through biochemical and functional characterization of RASSF2 in leukemia models, we establish that a SARAH domain-dependent interaction with Hippo kinases MST1 and MST2 is absolutely essential for stabilization of RASSF2 protein and for its tumor-suppressive function against RUNX1-ETO-mediated leukemogenesis. A proximity-based biotin labeling approach demonstrates that RASSF2 forms a discrete complex with MST1 and MST2 that is independent of the canonical Hippo pathway in leukemia cells. Furthermore, we reveal an association with the Rac GTPase-specific guanine nucleotide exchange factor, DOCK2, and demonstrate a functional role for RASSF2 in contributing to Rac GTPase activation in AML. These findings link RASSF2 with an emerging function of Hippo kinases in regulating Rac GTPase activity through DOCK family proteins in the context of normal and malignant hematopoietic cells.

## Materials and methods

Please see Supplementary Methods for additional experimental details.

### siRNA nucleofection

RUNX1-ETO and control-targeting siRNAs, as previously described^26^, were synthesized and purchased from GE Dharmacon (Lafayette, CO). Nucleofections were performed by addition of 2 μl siRNA to 100 μl cells (1.5 × 10^7^/ml) in Amaxa buffer V using program P-019 in an Amaxa nucleofector (Lonza, Cologne, Germany).

### Serial replating/colony formation assay

Serial replating/colony formation assays were performed essentially as described previously^27^, but with use of MSCV-PGK-Neo_R_ or MSCV-RUNX1-ETO-PGK-Neo_R_ retroviral expression vectors, and MSCV-IRES-Puro_R_ or MSCV-Rassf2-IRES-Puro_R_ (and variants), together.

### RUNX1-ETO9a/Rassf2 primary leukemia model

Transfections of HEK293T cells were conducted by combining 2.5 μg of MSCV-IRES-GFP or MSCV-RUNX1-ETO9a-IRES-GFP retroviral expression vector, 2.5 μg of MSCV-IRES-tdTomato or MSCV-Rassf2-IRES-tdTomato, along with packaging components. For transduction, primary E16.5–E18.5 murine fetal liver cells were resuspended in supplemented retroviral supernatant at densities of 2–3 × 10^6^ cells/ml and centrifuged (2000 × *g*) in six-well plates at 32 °C for 3 h (Allegra X-12R centrifuge, Beckman Coulter), followed by overnight culture at 37 °C. Two consecutive retroviral transductions were performed in this manner on subsequent days. Transduction efficiencies were measured (GFP+ and tdTomato+ frequencies) by flow cytometry. Transduced cell populations were flow sorted and then pooled with untransduced “helper” fetal liver cells at a ratio of 1:6. For the primary leukemia model, 150,000 total cells per mouse were then transplanted into lethally irradiated (9.5 Gy) recipients and leukemia was monitored. Recipients were randomly allocated to experimental groups with equal distribution based on sex. Recipient mice found to develop GFP-negative/non-myeloid malignancies were excluded from analysis. Investigators were not blinded during group allocation or assessment of experimental outcomes.

### Peripheral blood collection and analysis

Peripheral blood samples (approximately 100 μl per mouse) were collected and analyzed exactly as described previously^28^.

### Plasmids and molecular cloning

Murine Rassf2 cDNA was purchased from Open Biosystems (#MMM1013-9202192). Human RASSF2 cDNA vector was kindly provided by Dr. Geoffrey J. Clark. CMV-MCS-BioID2-HA-Neo^R^ (Addgene Plasmid #74224), CMV-MCS-13xLinker-BioID2-HA-Neo^R^ (Addgene Plasmid #80899), and CMV-myc-BioID2-MCS-Neo^R^ (Addgene Plasmid #74223) were kindly provided by Kyle Roux. pJ3H-MST1 (Addgene Plasmid #12203) and pJ3H-MST1-K59R (Addgene Plasmid #12204) expression plasmids were kindly provided by Jonathan Chernoff. Relevant cDNA inserts were inserted into MSCV-based retroviral expression vector backbones by PCR cloning. RASSF2 and MST1 truncation mutant cDNA fragments were generated by overlap PCR.

### Cycloheximide chase assay

Forty-eight hours following transfection with indicated constructs, HEK293T cells were washed and seeded in six-well plates containing 25 μM cycloheximide (Sigma, St. Louis, MO) and cultured for indicated lengths of time before harvesting lysates for protein analysis. Where applicable for proteasome experiments, 10 μM lactacystin (Sigma, St. Louis, MO) was also added to culture media.

### PAK1-based pull-down assay for active Rac-GTP measurement

Rac1 GTPase Activation Assay (Cell Biolabs # STA-401-1, San Diego, CA) was performed essentially as described by manufacturer’s instructions.

### Proximity-based biotin labeling and protein identification

AML cell lines stably expressing RASSF2-BioID2 or Control-BioID2 cDNA were established via retroviral transduction followed by 5 days of selection (1.5 µg/ml) with puromycin. Stable lines were then cultured in media (RPMI, 10% FBS, 1% Pen/Strep) supplemented with 50 µM biotin for 48 h (approximately one population doubling). Approximately 20 million cells per sample were washed, lysed, and subjected to streptavidin-based co-immunoprecipitation for identification of proximal proteins using the BioID2 method as described in Roux et al.^29^. Mass spectrometry was performed by the Sanford Burnham Prebys Proteomics Shared Resource.

### Differential expression analyses

Cell lysis and RNA isolation were performed using Trizol reagent (ThermoFisher Scientific, #15596026) according to the manufacturer’s instructions; isolated RNA was aliquoted and stored at −80 °C. Library preparation and sequencing were performed by Novogene (Sacramento, CA). For differential gene expression, six independent shRASSF2 replicates (three each, two shRNAs) are compared against three independent shCTRL replicates. Differentially expressed genes (DESeq2) are defined by:log2(fold-change) > 1.0 or <−1.0, and –log10(adjusted *p* value) > 3.0. Gene ontology term enrichment within the differentially expressed genes from TCGA AML patient cohorts was performed using ClusterProfiler^30^.

### Bone marrow mononuclear cell collection

Bone marrow mononuclear cell isolation and enrichment have been described previously^28^.

### Cell lines

Human AML cell lines Kasumi-1, SKNO-1, THP-1, MV4-11, U937, NB-4, K562, and HEK293T cells were purchased from ATCC and maintained as low passage stocks in the lab. OCI-AML3 AML cell line was kindly provided by Suming Huang (University of Florida, Gainesville, FL).

### Mice

All animal protocols were approved by the UCSD Institutional Animal Care and Use Committee (IACUC). Mice were housed in standard conditions in accordance with IACUC guidelines. C57BL/6J (stock #000664), Vav1-Cre (stock #008610)^31^ transgenic, and *Stk4*- and *Stk3-*floxed (stock #017635)^32^ mice were obtained from The Jackson Lab. Genotyping for floxed alleles and Cre transgenes was conducted as described on The Jackson Lab website.

## Results

### *RASSF2* is a transcriptionally repressed target of the RUNX1-ETO fusion protein in t(8;21) AML

**Fig. 1:**
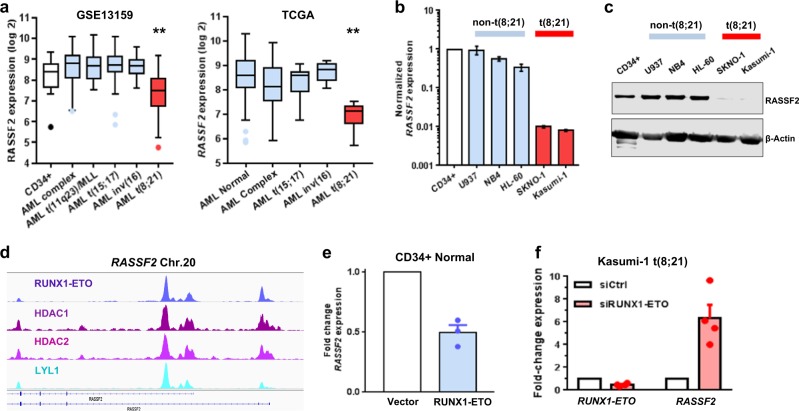
*RASSF2* is a transcriptionally repressed target of RUNX1-ETO in t(8;21) AML. **a** Normalized *RASSF2* expression from two independent cohorts of AML patients (GSE13159 (ref. ^33^), left and TCGA^1^, right). For GSE13159, CD34+ cells from healthy donors (*n* = 18) are indicated in white, non-t(8;21) AML patients (*n* = 192) are indicated in blue, and t(8;21) AML patients (*n* = 60) are indicated in red. For TCGA, non-t(8;21) AML patients (*n* = 164) are indicated in blue and t(8;21) AML patients (*n* = 7) are indicated in red. Data are presented as Tukey boxplots with outliers indicated by individual points. **p < 0.01, ANOVA followed by post hoc Tukey test. **b** RT-qPCR data showing *RASSF2* expression in non-t(8;21) (blue) and t(8;21) (red) AML cell lines. Data are normalized relative to healthy CD34+ cord blood cells and presented as mean ± s.e.m. of three experiments. **c** Western blot showing protein from whole-cell lysates as indicated, data are representative of three experiments. **d** ChIP-seq tracks showing binding of indicated transcription/epigenetic factors within the *RASSF2* genomic locus in the Kasumi-1 t(8;21) AML cell line. **e** Normalized RT-qPCR data showing fold-change *RASSF2* expression in cord-blood CD34+ cells 72 h post-transduction with empty or RUNX1-ETO expression vectors. Data are mean ± s.e.m. of three experiments (indicated by points). **f** Normalized RT-qPCR data showing fold-change *RASSF2* expression in Kasumi-1 cells 72 h post-nucleofection with control or RUNX1-ETO targeting siRNA. Data are mean ± s.e.m. of four experiments (indicated by points).

Using a mouse model of t(8;21) AML, we previously identified *Rassf2* as a gene that is transcriptionally downregulated greater than 20-fold in RUNX1-ETO expressing leukemic blasts compared to normal hematopoietic progenitors^13^. We therefore set out to study RASSF2 in the context of myeloid leukemia development. Consistent with an initial report^23^, we found *RASSF2* transcript expression to be most abundant in normal human peripheral blood tissue (Supplementary Fig. 1a), supportive of important normal functions in hematopoietic cell populations. To follow-up the murine leukemia study we assessed *RASSF2* transcript expression in two cohorts of AML patients (GSE13159 (ref. ^33^) and TCGA^1^, Fig. 1a). Consistent with the mouse model, *RASSF2* is uniquely downregulated in t(8;21) AML patients relative to other AML subtypes and healthy-donor CD34+ hematopoietic cells. We also found both RASSF2 mRNA (Fig. 1b) and protein (Fig. 1c) expression to be drastically downregulated (~10–50 fold) specifically in t(8;21) AML cell lines relative to non-t(8;21) AML cell lines and healthy human hematopoietic CD34+ cells. We next sought to determine whether this transcriptional repression was directly mediated by the RUNX1-ETO oncofusion protein in t(8;21) AML^6^. Supporting this hypothesis, both the *RASSF2* promoter CpG island^34^ and a downstream alternative transcription start site demonstrated chromatin co-occupancy of RUNX1-ETO, repressive histone deacetylases (HDAC1/2), and RUNX1-ETO transcription factor complex component LYL1 (ref. ^7^), in t(8;21) AML cells (Fig. 1d). These genomic regions are bound by the major hematopoietic transcription factors RUNX1, ERG, and FLI1 in healthy human CD34+ cells^35^, demonstrating these as major regulatory elements for controlling *RASSF2* expression during hematopoiesis (Supplementary Fig. 1b). Retroviral transduction of cord-blood-derived human CD34+ cells with a RUNX1-ETO expression vector was sufficient to reduce *RASSF2* transcript (Fig. 1e). Similarly, *RUNX1-ETO* knockdown using siRNA targeted to the fusion site^26^ was sufficient to increase *RASSF2* mRNA expression in a t(8;21) AML cell line (Fig. 1f). These findings are consistent with published studies in which *RASSF2* is reproducibly one of the most dynamically upregulated genes following *RUNX1-ETO* knockdown^7,8,36^. Together, these results demonstrate that *RASSF2* is differentially expressed across AML subtypes and is a direct transcriptional target of the RUNX1-ETO oncofusion protein in t(8;21) AML.

### Transcriptional repression of *RASSF2* contributes to the t(8;21)-associated gene expression signature in AML

**Fig. 2:**
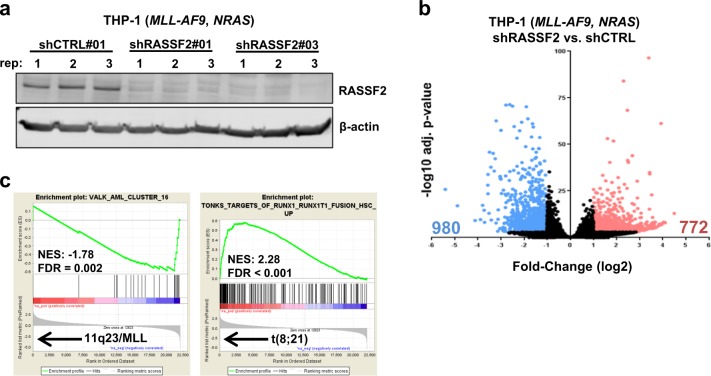
Transcriptional repression of *RASSF2* contributes to the t(8;21)-associated gene expression signature in AML. **a** Western blot showing RASSF2 protein in THP-1 cells following lentiviral transduction with indicated shRNAs. Data for nine independent replicates (three shCTRL, six shRASSF2) that were submitted for RNA-sequencing analysis are included. **b** Volcano plot of RNA-sequencing analysis depicting fold-change expression and significance for all detected genes in human THP-1 cells with stable transduction of two independent RASSF2-targeting shRNAs (shRASSF2, three replicates each) compared to control-targeting shRNA (shCTRL, three replicates). Significantly changed genes are highlighted (downregulated, blue; upregulated, red). **c** Gene-set enrichment plots as indicated derived from gene expression analysis in **b**. NES normalized enrichment score, FDR false discovery rate.

To further understand the impact of differential transcriptional regulation of *RASSF2* in AML, we performed gene expression profiling in a non-t(8;21) AML cell context following shRNA-mediated depletion of *RASSF2* transcript (Fig. 2a). *RASSF2* knockdown in MLL-rearranged THP-1 AML cells profoundly altered the global transcriptional signature (Fig. 2b, Supplementary Table 1). Remarkably, gene-set enrichment analysis (GSEA)^37^ revealed *RASSF2* knockdown to cause loss of a gene expression signature specifically associated with AML patients harboring *MLL* rearrangements^38^, and gain of a gene expression signature defined by expression of the t(8;21) oncofusion protein RUNX1-ETO^39^ (Fig. 2c). This observation highlights the contribution of a single-gene transcriptional repression event to overall AML subtype-specific downstream transcriptional output, and supports a model in which at least part of the RUNX1-ETO upregulated gene expression signature is mediated through indirect signaling consequences caused by transcriptional repression of key target genes. Furthermore, this overlap in gene expression signatures with a defined RUNX1-ETO expression signature in primary cells highlights the relevance of continued study of RASSF2 in the context of t(8;21) AML.

### Re-expression of RASSF2 is tumor-suppressive specifically in t(8;21) AML

**Fig. 3:**
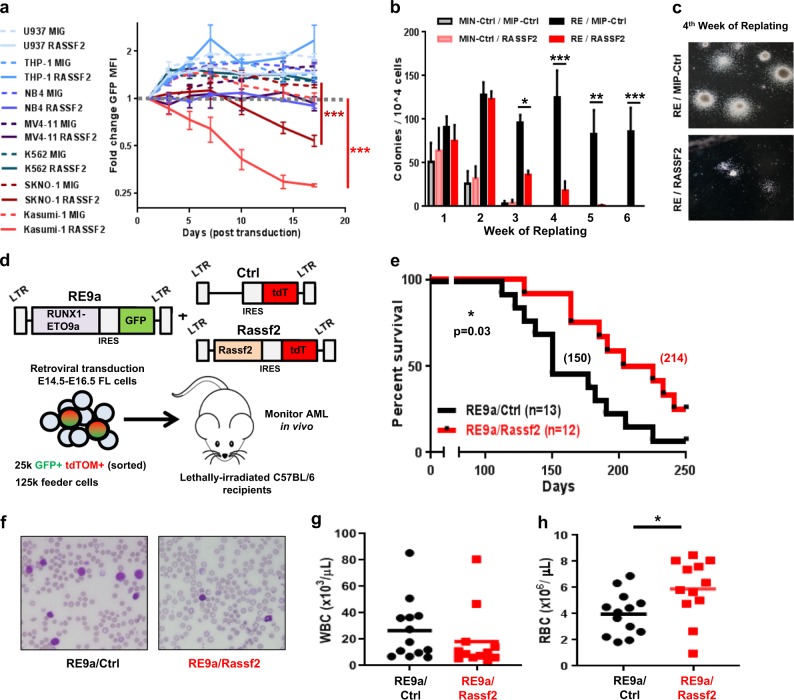
Re-expression of RASSF2 is tumor suppressive specifically in t(8;21) AML. **a** Fold-change in population mean GFP fluorescence intensity (MFI) over time was measured by flow cytometry in the indicated cell lines following transduction (efficiencies ~50–60%) with retroviral MSCV-IRES-GFP (MIG), or MSCV-RASSF2-IRES-GFP (RASSF2) vectors. Data are mean ± s.e.m. of four experiments. ***p < 0.001, two-tailed Student’s *t*-test performed at day 17 for cell lines in which GFP MFI of one vector-transduced population dropped below initial measurement value (day 1 post-transduction), which is indicated by dashed gray line. **b** Number of colonies (per 10,000 plated cells) for each week of serial replating of primary murine bone marrow cells transduced with vectors as indicated. Data are mean ± s.e.m. of five experiments. *p < 0.05, **p < 0.01, ***p < 0.001, two-tailed Student’s *t*-test. See also Supplementary Fig. 2b. **c** Representative images of colonies from fourth week of replating (**b**) for indicated populations. **d** Schematic for primary RUNX1-ETO9a retroviral transduction/transplantation murine model of t(8;21) AML with re-expression of *Rassf2* (RE9a/Rassf2) or vector control (RE9a/Ctrl). **e** Survival analysis for experiment described in **d**; significance determined by log-rank (Mantel-Cox) test. **f** Representative peripheral blood smears of indicated mice from experiment described in **d** at 125 days post-transplantation. **g**, **h** Peripheral (**g**) white blood cell (WBC) and (**h**) red blood cell (RBC) counts of indicated mice from experiment described in **d** at 125 days post-transplantation, solid lines indicate population mean. *p < 0.05, two-tailed Student’s *t*-test.

We next explored the functional consequences of transcriptional repression of *RASSF2* to leukemogenesis. Using retroviral GFP-based reporter vectors, we screened the effect of RASSF2 expression across a panel of AML cell lines, using change in GFP fluorescence intensity over time as a functional readout for cell proliferation. Only upon expression in the t(8;21) AML cell lines, Kasumi-1 and SKNO-1, did RASSF2 impart a competitive growth disadvantage relative to control-transduced (GFP only) populations (Fig. 3a). Importantly, assessment of protein expression in RASSF2-transduced t(8;21) cell populations showed that re-expression occurred in a physiologically relevant manner and was not overexpressed compared to endogenous amounts observed in non-t(8;21) AML cell lines (Supplementary Fig. 2a). These results suggest that RASSF2 is a context-specific, rather than general, tumor suppressor protein in myeloid leukemia. We next assessed the ability of RASSF2 to suppress RUNX1-ETO-mediated leukemic transformation of primary murine hematopoietic progenitors using a serial replating/colony formation assay (Supplementary Fig. 2b). Re-expression of RASSF2 was sufficient to block RUNX1-ETO-mediated long-term clonogenic self-renewal after 3–4 weeks (Fig. 3b, c). We also assessed RASSF2-mediated tumor suppression in vivo using a murine retroviral transduction and transplantation model of t(8;21) AML with the alternatively spliced leukemogenic protein variant, RUNX1-ETO9a (RE9a, Fig. 3d)^40^. Co-expression of *Rassf2* significantly delayed RE9a leukemia onset (median survival 214 vs 150 days) compared to an empty-vector control (Fig. 3e). The delay in leukemia onset was associated with reduced leukemic burden in the peripheral blood (Fig. 3f, g, Supplementary Figs. 2c, d) and less severe anemia (Fig. 3h, Supplementary Fig. 2e). Terminal assessment of moribund mice revealed AML development in both populations with no significant morphologic differences between cohorts (Supplementary Fig. 2f). These data reveal RASSF2 to be a physiologically relevant tumor suppressor capable of inhibiting t(8;21) AML development both in vitro and in vivo.

### RASSF2 function is independent of nucleo-cytoplasmic shuttling and oncogenic Ras signaling in hematologic cancer

We next sought to explore the mechanism of RASSF2 function in myeloid leukemogenesis. RASSF proteins possess no enzymatic activity and are primarily thought to function as scaffolds through their protein–protein interaction domains^15^. RASSF2 is reported to exert tumor-suppressive functions as a nucleo-cytoplasmic shuttling protein^41^. We could not, however, detect any evidence for nuclear localization of endogenous or exogenous RASSF2 in myeloid leukemia cell lines, while still replicating nucleo-cytoplasmic shuttling upon expression in HEK293T cells (Supplementary Figs. 3a–c). These results are consistent with data from the Human Protein Atlas^42^, in which RASSF2 is exclusively localized to the cytoplasmic/membranous compartments in hematopoietic cells (www.proteinatlas.org). Several RASSFs have been implicated in negative regulation of oncogenic Ras function through the Ras-association (RA) domain^14,15,23^. Activating point mutations in *NRAS* and *KRAS* are frequent in AML and often co-occur with t(8;21)^43^. Despite this, re-expression of RASSF2 in t(8;21) AML cells did not affect canonical Ras signaling through the MAPK/ERK or AKT pathways following stimulation with cytokines (Supplementary Fig. 3d). Furthermore, we found no correlation between *RASSF2* expression and the presence of oncogenic *RAS* mutations more broadly in AML patients (Supplementary Fig. 3e). These data warranted investigation of RASSF2 function through additional mechanisms.

### RASSF2-mediated tumor suppression is dependent on interaction with Hippo kinases MST1/2

**Fig. 4:**
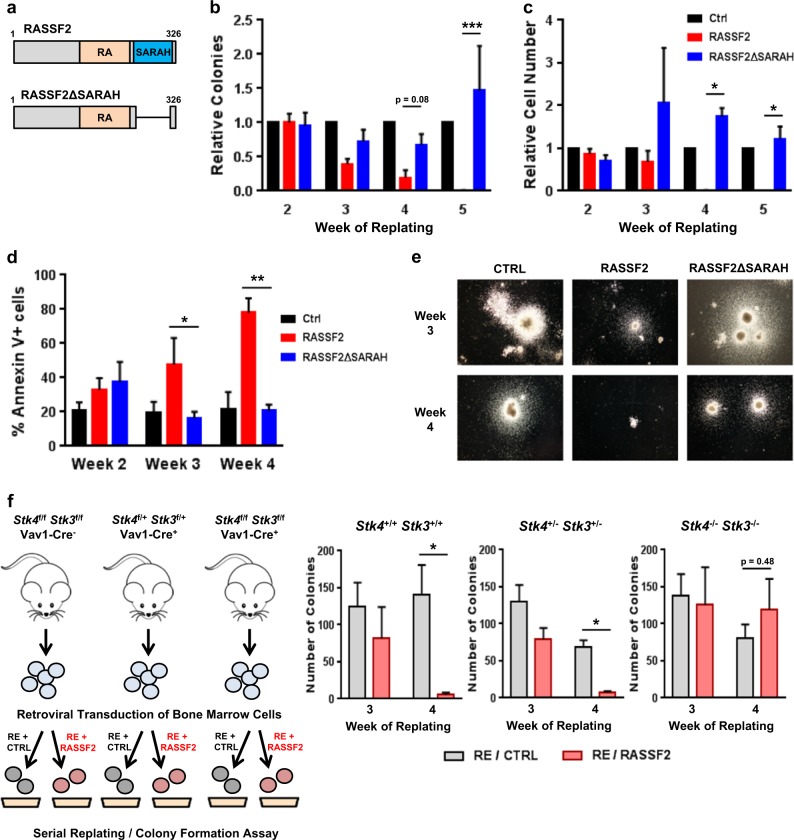
RASSF2-mediated tumor suppression is dependent on interaction with Hippo kinases MST1/2 in t(8;21) AML. **a** Schematic of RASSF2 proteins expressed in experiments **b–e**, RA, Ras-association domain, SARAH, Salvador-Rassf-Hippo domain. **b** Colony number (per 10,000 cells plated), **c** total viable cell number (per 10,000 cells plated), **d** frequency of Annexin-V+ apoptotic cells, and **e** representative images of colonies for RUNX1-ETO serial replating/colony formation assay with co-transduction of indicated vectors. Data are normalized relative to empty-vector control-transduced populations and presented as mean ± s.e.m. of four independent experiments. *p < 0.05, **p < 0.01, ***p < 0.001, two-tailed Student’s *t*-test. **f** RUNX1-ETO primary murine serial replating/colony formation assay was performed in mice of three indicated genotypes, with expression of RASSF2 or empty-vector control, as shown in schematic (left). Colony numbers (per 10,000 cells plated) are shown for third and fourth week of replating for each genotype (right). Data are mean ± s.e.m. of three experiments. *p < 0.05, two-tailed Student’s *t*-test.

All six classical RASSFs contain a highly-conserved C-terminal Salvador-Rassf-Hippo (SARAH) domain that is unique to protein components of the Hippo signaling pathway and mediates homo- or hetero-dimerization between SARAH domain-containing proteins^15^. Structural and biochemical analyses suggest that the mammalian Hippo kinases, MST1 and MST2, primarily exist as SARAH-mediated heterodimers with RASSF proteins under basal cellular conditions^44^. To determine whether interaction with MST1/2 was required for RASSF2 function, we generated a RASSF2 deletion mutant lacking the SARAH domain (RASSF2ΔSARAH) for use in the serial replating/colony formation assay (Fig. 4a, Supplementary Fig. 2b). RASSF2ΔSARAH completely lost the ability to suppress RUNX1-ETO leukemic transformation and induce apoptosis in RUNX1-ETO-expressing cells (Fig. 4b–e). To further confirm the requirement of Hippo kinases for RASSF2-mediated tumor suppression we generated mice with conditional gene inactivation of both MST1 (*Stk4*) and MST2 (*Stk3*) in hematopoietic cells (Vav1-Cre). By co-expression of RUNX1-ETO and RASSF2 in the background of control (Vav1-Cre^−^), heterozygous (Stk4^f/+^Stk3^f/+^;Vav1-Cre^+^), and homozygous (Stk4^f/f^Stk3^f/f^;Vav1-Cre^+^) knockout mice, we found that the presence of MST1/2 was essential for the ability of RASSF2 to inhibit RUNX1-ETO leukemic transformation (Fig. 4f). These data revealed the importance of the Hippo kinase-RASSF2 interaction and we set out to further characterize this.

### RASSF2 is completely dependent on Hippo kinases for stabilization in vitro and in vivo

**Fig. 5:**
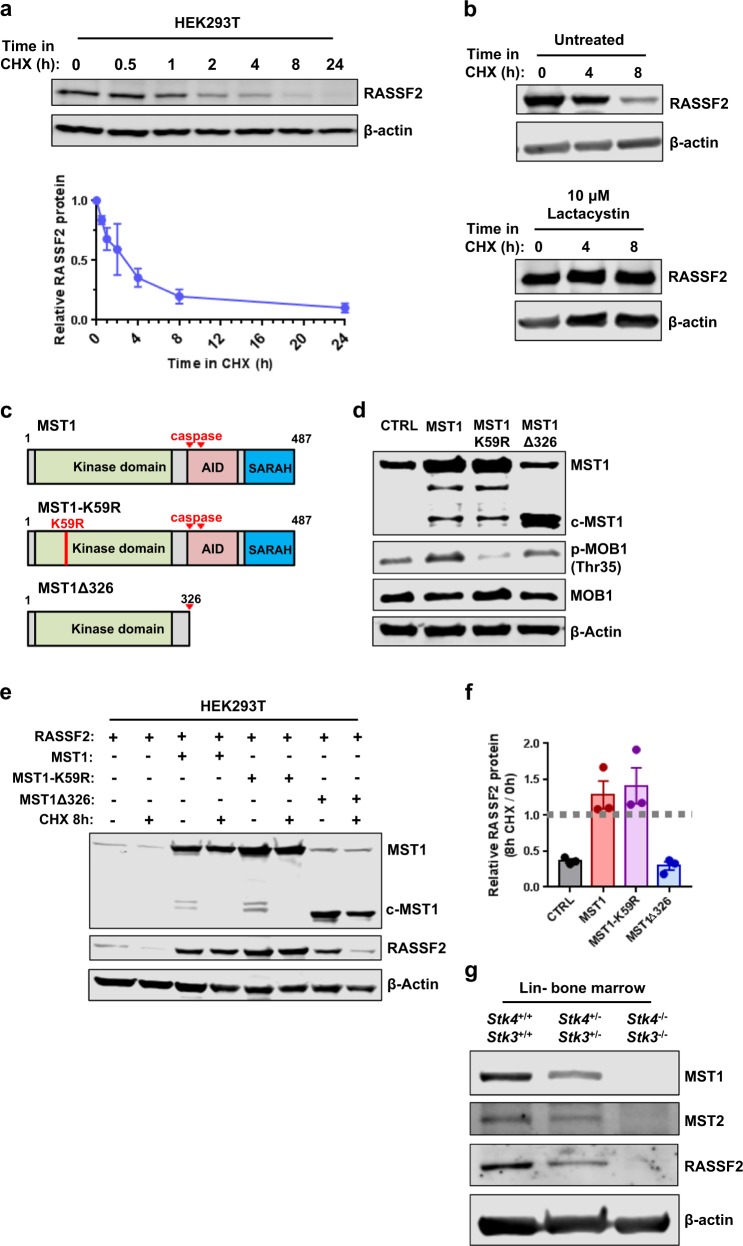
RASSF2 is completely dependent on stabilization by Hippo kinases in vitro and in vivo. **a** Western blot showing time-course of protein expression following addition of 25 μM cycloheximide (CHX) from HEK293T whole cell lysates beginning 48 h post-transfection with RASSF2 expression vector. Quantification of three experiments is shown below. **b** Western blot showing time-course of protein expression following addition of 25 μM cycloheximide (CHX) from HEK293T whole-cell lysates beginning 48 h post-transfection with RASSF2 expression vector, with (bottom) or without (top) the addition of 10 μM Lactacystin. Data are representative of three experiments. **c** Schematic of MST1 protein variants expressed in experiments **d–f**. K59R, substitution of arginine for lysine at amino acid residue 59 results in kinase-dead protein that functions as dominant negative; AID, auto-inhibitory domain; SARAH, Salvador-Rassf-Hippo domain. Red arrows represent endogenous caspase-cleavage sites. **d** Western blot showing protein from whole-cell lysates in the SKNO-1 t(8;21) cell line transduced with MST1-variant retroviral expression vectors as indicated. c-MST1 caspase-cleaved MST1. **e** Western blot showing protein from HEK293T whole-cell lysates 72 h post-transfection with indicated expression vectors, with or without the addition of 25 μM cycloheximide (CHX) for the final 8 h as indicated. Data are representative of three experiments. **f** Quantification of relative change in RASSF2 protein abundance with or without cycloheximide treatment from three experiments described in **e**. **g** Western blot showing protein from whole-cell lysates of Lineage-marker negative (Lin−) primary murine hematopoietic cells derived from bone marrow of mice of indicated genotypes. Cells from three mice per genotype are pooled for analysis.

Despite a modest stabilizing effect on MST1 and MST2, RASSF2 expression did not alter signaling through canonical Hippo pathway targets MOB1 and LATS1 (ref. ^25^) in t(8;21) AML cells (Supplementary Figs. 4a, b). These findings are consistent with another report in which RASSF2 expression did not affect canonical MST1/2 phosphorylation targets^24^. Therefore, we hypothesized that RASSF2-mediated tumor suppression against RUNX1-ETO-expressing cells may be dependent on SARAH domain-dependent stabilization by the Hippo kinases. Consistent with this, we found RASSF2 protein to have a relatively short half-life of 2–3 h in HEK293T cells in cycloheximide chase assays (Fig. 5a). This degradation was proteasome-dependent (Fig. 5b). Based on this, we further explored the kinetics of RASSF2 protein stability and its relationship with Hippo kinase MST1. To determine whether kinase activity was required for RASSF2 function we utilized a kinase-dead point-mutant protein, MST1-K59R (Fig. 5c). We also included a biologically relevant C-terminal truncation mutant of MST1 that mimics caspase-cleavage at amino acid 326 (MST1Δ326), which is widely accepted to exert increased proapoptotic kinase activity through removal of an auto-inhibitory domain (AID), while also losing its ability to dimerize with RASSF proteins through the SARAH domain (Fig. 5c)^45^. We confirmed MST1-K59R to act as a dominant negative kinase towards MOB1 phosphorylation (Fig. 5d). We then assessed the ability of MST1 and its protein variants to protect RASSF2 from proteasomal degradation. Consistent with being independent of kinase activity, MST1 and MST1-K59R, but not MST1Δ326, effectively protected RASSF2 from degradation following cycloheximide treatment (Fig. 5e, f). This revealed that the ability of MST1 to interact with RASSF2 through the SARAH domain, rather than its kinase activity towards canonical effectors, contributes to its function in myeloid leukemia cells. To confirm the relevance of this non-canonical Hippo kinase function in vivo we assessed protein expression in murine hematopoietic progenitor cells from conditional knockout mice described above, and found endogenous RASSF2 protein to be completely dependent on the presence of MST1/2 for stabilization (Fig. 5g). These data demonstrate that a previously unappreciated kinase-independent function of the Hippo kinases MST1/2 can contribute to regulation of cancer cell growth through SARAH domain-dependent stabilization of RASSFs.

### Proximity-dependent biotin labeling defines the RASSF2-proximal proteome and reveals a role in regulation of Rac GTPase activity in AML

**Fig. 6:**
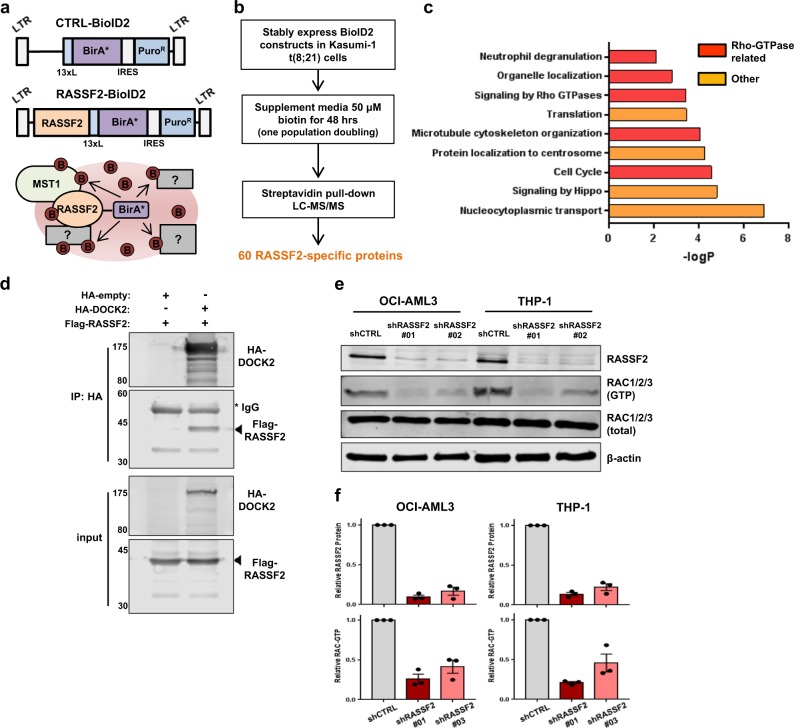
Proximity-dependent biotin labeling (BioID) identifies a role for RASSF2 in regulation of Rac GTPase activation in AML. **a** Schematic showing retroviral expression vectors and general assay principle for performing proximity-based biotin labeling and identification using an improved biotin ligase (BioID2, see Methods). **b** Strategy for performing proximity-based biotin labeling in Kasumi-1 t(8;21) cell line. Experiment was performed in three independent replicates that were submitted for mass-spectrometric analysis, and 60 unique high-confidence RASSF2-proximal proteins were identified across three replicates. **c** Bar graph showing significantly enriched Gene Ontology (GO) terms associated with the RASSF2-specific protein hits identified by proximity-based biotin labeling. **d**, Western blot showing interaction between HA-DOCK2 and Flag-RASSF2 as assessed in HEK293T cell lysates by immunoblotting following HA-immunoprecipitation (IP) ~48 h post-transfection with indicated constructs. Inputs are shown below. Molecular weight markers (kDa) are indicated at left. Data are representative of three experiments. **e** Immunoblotting for active (GTP-bound) Rac by PAK1 pull-down assay performed in two representative AML cell lines 4 days post-transduction with lentiviral shRNAs targeting control sequence (shCTRL) or *RASSF2* (shR2#1, shR2#2) as indicated. Data are representative of three experiments. **f** Quantification of experiments performed in **e**. Data are normalized relative to β-actin protein abundance and presented as mean ± s.e.m. of three experiments.

RASSF proteins lack enzymatic activity and instead mediate their functions through various protein–protein interactions. These potential interactions have not previously been characterized in the context of hematologic cancer. Our data revealed RASSF2 to suppress cancer growth in the context of t(8;21) AML so we set out to identify proteins/complexes associated with RASSF2 in leukemia cells. To do this, we employed a proximity-dependent biotin labeling (BioID) approach^29^ to identify physiologically relevant proximal proteins upon re-expression of RASSF2 in t(8;21) AML cells. We tested three different orientations for expression of the RASSF2-BioID2^46^ fusion cassette, and ultimately chose a C-terminal fusion separated with a linker peptide based on its retained ability to interact with and biotinylate MST1 as a positive control (Fig. 6a, Supplementary Figs. 5a–c). Affinity purification of biotinylated proteins in Kasumi-1 t(8;21) AML cells was performed using steptavidin-conjugated beads and samples were submitted for mass spectrometry analysis following stringent washing conditions (Fig. 6b, Supplementary Fig. 5d). After strict filtering based on the CRAPome^47^ we identified 60 unique RASSF2-specific proximal proteins across three independent replicates in Kasumi-1 cells (Supplementary Table 2). Hippo kinases MST1 and MST2 were the first- and sixth-ranked hits, respectively, highlighting the accuracy of this approach. Gene ontology (GO) analysis of the full list of proteins revealed RASSF2 to be associated with several pathways and cellular functions related to signaling by Rho-family GTPases (Fig. 6c). This proximal proteome includes general downstream Rho-effectors related to regulation of microtubule polymerization, including PPP1CC, NUDC, SEH1L, STMN1, STMN2, FKBP4, and MAP4, as well as proteins involved in specific-regulation of the localization and activation of Rac-family GTPases, DVL2, ANXA2, and DOCK2. No additional canonical Hippo pathway components or SARAH domain-containing proteins were identified, demonstrating the discrete nature of a Hippo kinase-RASSF2 non-canonical signaling complex in leukemia cells.

One specific identified protein of interest was the Rac1/2-specific atypical guanine nucleotide exchange factor (GEF), DOCK2. DOCK2 functions as an essential positive regulator of Rac1/2 activation in hematopoietic cells by catalyzing nucleotide exchange via a DHR2 domain^48^. We confirmed the interaction between RASSF2 and DOCK2 via co-immunoprecipitation experiments (Fig. 6d). Based on an association with DOCK2, we examined whether RASSF2 perturbation may have an effect on endogenous Rac GTPase activity in AML. Strikingly, lentiviral shRNA-mediated knockdown of *RASSF2* in myeloid leukemia cells with two independent shRNAs was sufficient to profoundly reduce endogenous Rac-GTP to ~ 30% of basal amounts (Fig. 6e, f). These data demonstrate a previously unappreciated link between Hippo kinases, RASSF2, and Rac GTPases in the context of AML.

**Fig. 7:**
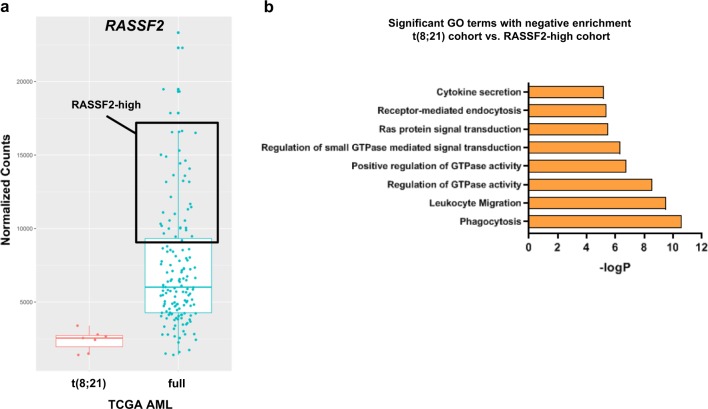
*RASSF2* expression is broadly predictive of Rac GTPase signal transduction in AML. **a** Normalized *RASSF2* expression (counts) in t(8;21) AML patient cohort (left) and full TCGA AML cohort (right) showing stratification of patients into two sub-cohorts for purpose of differential gene expression analysis: t(8;21) and RASSF2-high (defined as upper quartile by gene expression). **b** Bar graph showing top-nine significantly enriched Gene Ontology (GO) terms, with negative enrichment (downregulation) in t(8;21) cohort, as determined by differential gene expression analysis of t(8;21) AML patient cohort vs. RASSF2-high AML patient cohort as defined in **a**.

To determine whether this mechanistic finding could be applied more broadly in AML, we utilized published RNA-sequencing data (TCGA cohort)^1^. Based on the marked RUNX1-ETO-dependent transcriptional repression of *RASSF2* (Fig. 1), we hypothesized that comparing gene expression between t(8;21) AML patients and patients with high *RASSF2* transcript expression (RASSF2-high) would reveal underlying differences in cellular biology and signal transduction pathways. Indeed, differential expression analysis and subsequent GO enrichment revealed the top-two most significantly e cellular processes in t(8;21) AML patients (relative to the RASSF2-high cohort) to be signatures of phagocytosis and leukocyte migration (Fig. 7a, b), which are both well-established to be dependent on Rac GTPases^49,50^. Furthermore, several additional significant GO terms directly indicated reduced Rho-family GTPase activation in t(8;21) AML patients compared to the RASSF2-high cohort (Fig. 7b). Together these data demonstrate a meaningful biologic role for RASSF2 expression in regulating Rac GTPase signal transduction and cellular processes in AML.

## Discussion

In summary, we identify RASSF2 as a critical transcriptionally repressed target gene of the RUNX1-ETO fusion protein in t(8;21) AML. RASSF2 is abundantly expressed in normal hematopoietic cell populations and the majority of AML subtypes, but is specifically downregulated in the presence of RUNX1-ETO. RUNX1-ETO directly binds at multiple sites within the *RASSF2* genomic locus, and depletion of RUNX1-ETO reproducibly results in upregulation of *RASSF2* transcript. This transcriptional repression has downstream functional consequences to t(8;21)-associated leukemogenesis, as *RASSF2* knockdown in a non-t(8;21) AML context is sufficient to reproduce an established RUNX1-ETO gene expression signature. Importantly, re-expression of RASSF2 in a physiologically relevant manner specifically inhibits t(8;21) leukemia cell growth, in both in vitro and in vivo assays. These findings not only demonstrate an important role for this specific transcriptional repression event, but also highlight a context-specific nature of RASSF2 in suppression of AML.

RASSF2 and other RASSFs have previously been linked with oncogenic Ras signaling, through the presence of their Ras-association (RA) domain, in a variety of cancer contexts; however, we found no evidence of this in AML. Similarly, we did not find evidence of Ras pathway-associated proteins or GTPases in our proximal biotin labeling experiments. These findings are consistent with Ras-centric proteomic studies that have not identified RASSF proteins as endogenous interactors of either wild type or mutant Ras proteins^51^. Instead, we here define the RASSF2-proximal proteome in leukemia cells and demonstrate the critical importance of its endogenous interaction with Hippo kinases, MST1 and MST2, through the C-terminal SARAH domain. This interaction is required to protect RASSF2 from proteasomal degradation, which we find evidence for both in vitro and in vivo. Expression of full-length MST1 or a kinase-dead variant (MST1-K59R), but not a C-terminally truncated protein that mimics physiologic caspase-cleavage and lacks the SARAH domain (MST1Δ326), is sufficient for stabilization of RASSF2. These findings suggest the potential for an underappreciated non-canonical signaling mechanism of MST1 and MST2 that functions independently of kinase activity, and instead relies on SARAH domain-dependent stabilization of RASSF2 or other RASSF proteins. Future studies may wish to further characterize this relationship in human cancer.

Through proximity-based biotin labeling (BioID2) and co-immunoprecipitation experiments, we find evidence that RASSF2 interacts with DOCK2 and positively contributes to regulation of Rac GTPase activity mediated by DOCK2 in AML cells. Importantly, several recent studies have revealed a mechanistic link between the Hippo kinases and regulation of Rac GTPase activity in hematopoietic cell populations. For example, MST1 and MST2 have been linked with positive regulation of Rac during the generation of reactive oxygen species and bactericidal activity in macrophages^52^. MST1 and MST2 were also recently identified in complex with the DOCK family Rac-GEF, DOCK8, in regulatory T cell populations, where they were found to be essential for Rac activation in response to IL-2 stimulation^53^. Recently, rare families harboring homozygous germline inactivating mutations in *STK4* (MST1) have been identified with clinical presentation of a lethal combined immunodeficiency syndrome and autoimmune reactions^54,55^. Remarkably, there is striking phenotypic overlap between individuals with *STK4* deficiency, and individuals with immunodeficiencies caused by homozygous inactivation of the atypical Rac-GEFs, *DOCK2* and *DOCK8* (refs. ^56,57^). This is compelling functional evidence in humans supporting a mechanistic link between Hippo kinases and sustained Rac GTPase activation in immune cell populations. Our data suggest an important contribution by RASSF2 in cooperation with DOCK2 to this process in myeloid leukemia cells, which we reveal to have important consequences in AML.

We find that knockdown of RASSF2 in a non-t(8;21) cell context potently reduces GTP-bound Rac, and conversely, Rac GTPase-deficient gene expression signatures are readily apparent in t(8;21) AML patients as compared to a RASSF2 highly expressing cohort. Although the precise mechanisms through which RASSF2 is capable of suppressing t(8;21) AML cell growth remain incompletely understood, our data suggest a role for Rac GTPase signal transduction in this process. The downstream outputs of activated Rac signaling vary depending on the cell type or cancer context and include (1) cell cycle regulation; (2) signaling through JNK/SAPKs, p38-MAPKs, PAKs, the NF-kB pathway, WASP/WAVE complexes, and the BCL-2 family; (3) regulation of actin dynamics during leukocyte migration and phagocytosis; and (4) NADPH oxidase activation for reactive oxygen species (ROS) generation^58,59^. Interestingly, Rac GTPases have previously been shown to be essential for sustained leukemia cell growth in AML driven by different oncogenes, such as in *MLL*-rearranged leukemias^60^, further demonstrating the context-specific nature of RASSF2 function in AML. We propose that one or more of these Rac GTPase signal transduction pathways are uniquely tumor suppressive in the context of t(8;21) AML, and therefore contribute to the importance of transcriptional repression of *RASSF2* for this specific leukemia subtype. For example, RUNX1-ETO has previously been shown to independently induce ROS and senescence-like growth arrest^61^, hinting that additional ROS generation through Rac/NADPH oxidase activity may have detrimental effects on t(8;21) AML cell growth. Future studies may seek to delineate which of these pathways downstream of Rac activation are critical for suppression of t(8;21) AML cell growth. Exploration of potential synthetic lethal genetic interactions in Rac-deficient cells may also be worth consideration for treatment of t(8;21) AML. The findings herein represent a significant step towards a more detailed understanding of mechanisms that uniquely oppose leukemic transformation by the RUNX1-ETO fusion protein in t(8;21) AML. Furthermore we identify a previously unappreciated function of RASSF2 in regulating Rac GTPase signal transduction that is more broadly applicable in AML.

## Supplementary information

Supplementary Material

Supplementary Table 1

Supplementary Table 2
