# Supplementary material for: The RUNX1-ETO target gene RASSF2 suppresses t(8;21) AML development and regulates Rac GTPase signaling
Source: Blood Cancer J. 2020 Feb 6;10(2):16. doi: 10.1038/s41408-020-0282-9 (PMC7005177; doi:10.1038/s41408-020-0282-9)
Supplement: Supplementary file 1 — Supplementary Material [file 41408_2020_282_MOESM1_ESM.pdf]

Supplementary Material

Supplementary Methods

Generation of Viral Supernatant and Transduction

For retroviral transduction experiments: Transfections of HEK293T cells were conducted by combining 5 µg of MSCV-IRES-GFP/Puro vectors 5 µg of packaging vector (pCL-10A1), and 40 µl of polyethylenimine (PEI) in 1 mL of Opti-MEM reduced serum medium (Gibco, #31985-070). Approximately 16 hours post-transfection, media was aspirated, cells were washed once in PBS, and 6 mL fresh IMDM (Gibco, #12440-053) supplemented with 10% fetal bovine serum was added to each plate. 24 hours following media change, IMDM media containing retroviral particles was collected, passed through a 0.45 µm syringe filter, pooled, and supplemented with recombinant cytokines mIL-3 (10 ng/mL), hIL-6 (10 ng/mL), and mSCF (20 ng/mL), and polybrene (4 µg/mL). For retroviral transduction, HSC-enriched bone-marrow mononuclear cells harvested from mice were resuspended in this supplemented retroviral supernatant at densities of 2-3 x 10^6 cells/mL and centrifuged (2,000x *g*) in 6-well plates at 32°C for 3 hours (Allegra X-12R centrifuge, Beckman Coulter); followed by overnight culture at 37°C. Two consecutive retroviral transductions were performed in this manner on subsequent days. Transduction efficiency was measured (GFP+ frequency) by flow cytometry the morning following the second centrifugation (immediately prior to transplantation into recipient mice). Low retroviral titers were utilized, which were typically measured at ~1.5-2% GFP+ following two rounds of transduction.

For lentiviral transduction of shRNA constructs: Transfections of HEK293T cells were conducted by combining 5 µg of psPAX2, 2.5 µg of pMD2.G, and 3 µg of respective pLKO.1-based shRNA vector, and 44 µl of polyethylenimine (PEI) in 1 mL of Opti-MEM reduced serum medium (Gibco, #31985-070). Approximately 16 hours post-transfection, media was aspirated, cells were washed once in PBS, and 10 mL fresh RPMI supplemented with 10% fetal bovine serum was added to each plate. 24 hours following media change, RPMI media containing lentiviral particles was collected, passed

through a 0.45 μm syringe filter, pooled, and supplemented with polybrene (final working concentration: 4 μg/mL). For lentiviral transduction, THP-1 cells were resuspended in this supplemented retroviral supernatant at densities of ~ 0.5 x 10^6 cells/mL and centrifuged (2,000x *g*) in 6-well plates at 32°C for 3 hours (Allegra X-12R centrifuge, Beckman Coulter); followed by overnight culture at 37°C. Two consecutive transductions were performed in this manner on subsequent days. 24 hours following second transduction cells were resuspended in 1.0 μg/mL puromycin, and selected for 72 hours, at which point viable drug-resistant cells were enriched via FICOLL gradient. Post-selection cells were maintained in growth media supplemented with 0.5 μg/mL puromycin.

siRNA Nucleofection

Lyophilized siRNAs were resuspended in siRNA buffer (20 mM KCl, 6 mM HEPES pH 7.5, 0.02 mM MgCl).

siRUNX1-ETO (sense strand):

5'-CCUCGAAAUCGUACUGAGAAG-3'

Non-targeting siCTRL (sense strand):

5'-CGUACGCGGAAUACUUCGATT-3'

qPCR Analysis

Cell lysis and RNA isolation were performed using Trizol reagent (ThermoFisher Scientific, #15596026) according to manufacturer's instructions. cDNA was prepared from 0.5 – 1 μg RNA using qScript cDNA Supermix (Quanta, #95048) according to manufacturer's protocol. Quantitative PCR was performed using KAPA SYBR Fast 2X Master Mix (KAPA Biosystems, #KK4618) according to manufacturer's protocol, in 20 μl reactions, each performed in technical duplicates. qPCR reactions were performed using a BioRad CFX Connect instrument. Data analysis was performed using a standard delta-delta Ct method relative to the geometric-mean of two reference genes, *GAPDH* and *POLR2A.*

Apoptosis Measurement

Apoptotic cell death was monitored by flow cytometry using the APC Annexin V apoptosis kit with 7-AAD (BioLegend, San Diego, CA) as described by manufacturer's protocol.

Western Blotting and Co-Immunoprecipitation

Samples were lysed in ice-cold NP-40 lysis buffer (50 mM Tris pH 8.0, 150 mM NaCl, 1% NP-40) supplemented with protease inhibitor (Roche, #11873580001) and phosphatase inhibitor (Roche, #4906845001). Lysates were cleared by centrifugation at 11,000 xg for 10 minutes at 4°C, and denatured in 2x loading buffer (0.125 M Tris-HCl pH 6.8, 4% SDS, 10% β-mercaptoethanol, 20% glycerol, 0.004% bromophenol blue). For co-immunoprecipitation experiments approximately 500 µg lysate was combined with 40 µl of anti-Flag M2 agarose beads (Sigma #M8823, St. Louis, MO), anti-HA beads (Fisher Scientific # 22-037-959, Waltham, MA) and incubated at 4°C with rotating overnight. For DOCK2 protein pull-down experiments without using tag, lysates were first pre-cleared by incubation with 40 µl protein G-sepharose bead slurry (Invitrogen, Carlsbad, CA) for one hour with rotating at 4°C, followed by washing and then combining 500 µg cell lysates with protein G-sepharose bead slurry and 2 µg monoclonal DOCK2 (E-7) antibody (Santa Cruz Biotechnology, Dallas, TX). The following morning beads were washed 4x with ice-cold NP-40 lysis buffer via centrifugation at 11,000 xg. Washed beads were then resuspended in 60 µl 2x loading buffer and denatured prior to loading. Immunoblotting was performed using the Li-Cor Odyssey infrared imaging instrument. Post-acquisition image analysis and cropping was performed using Li-Cor Image Studio Lite (V 5.2.5) software.

PAK1-based Pull-down Assay for Active Rac-GTP Measurement

Briefly, lysates from approximately 6 x 10^6 cells were prepared as described above but with 1X GTPase assay/lysis buffer (25 mM HEPES, pH 7.5, 150 mM NaCl, 1% NP-40, 10 mM $MgCl_2$, 1 mM

EDTA, 2% Glycerol). After clearing, 5% of cell lysates were separated and denatured in 2X loading buffer for use as input protein measurements. For pull-down, remaining cell lysates were combined with 36 µL PAK1 PBD agarose beads (#STA-411) and incubated at 4°C with rotating either for 3 hours (human AML cell lines) or overnight (HEK293T cells). Following this, beads were washed 3x with ice-cold 1X Assay/Lysis buffer via centrifugation at 11,000 xg. Washed beads were then resuspended in 50 µl 2x loading buffer and denatured prior to loading. Immunoblotting was performed using the Li-Cor Odyssey infrared imaging instrument. Post-acquisition image analysis and cropping was performed using Li-Cor Image Studio Lite (V 5.2.5) software. For measuring Rac-GTP in cells, pan-Rac antibody (see Antibody section), rather than anti-Rac1 monoclonal antibody provided by kit was used. Positive and negative assay controls using 100X GTPγS and 100X GDP were performed in parallel using excess cell lysates.

RNA-seq Analysis

Post-QC, mRNA from eukaryotic organisms is enriched using oligo(dT) beads. First, the mRNA is fragmented randomly by adding fragmentation buffer, then the cDNA is synthesized by using mRNA template and random hexamers primer, after which a custom second-strand synthesis buffer (Illumina) , dNTPs, RNase H and DNA polymerase I are added to initiate the second-strand synthesis. Second, after a series of terminal repair, ligation and sequencing adaptor ligation, the double-stranded cDNA library is completed through size selection and PCR enrichment. The qualified libraries are fed into HiSeq/MiSeq sequencers after pooling according to its effective concentration and expected data volume. Differential expression analysis was conducted by established methods using HISAT2 for human genome alignment (hg38), followed by featureCounts and DESeq2 for differential gene expression analysis.

Gene Ontology Analysis

Gene ontology analysis was performed using Metascape (metascape.org). Gene list of RASSF2-specific protein hits was uploaded and compared against full human genome list as background.

Flow Cytometry and Cell Sorting

Flow cytometric analysis was conducted using a BD FACSCanto instrument equipped with standard lasers (488 nm, 640 nm) and filters. Data collection was performed using BD FACSDiva software. Compensation was set up using appropriate single-stained controls and positive-staining gates were established using appropriate FMO controls. Post-acquisition data analysis was performed using FlowJo software (FlowJo, LLC). Flow sorting of bone-marrow mononuclear cells was performed on a FACSAriaII equipped with standard lasers (405 nm, 488 nm, 640 nm) and filters, and using a nozzle size of 85 μm. Stainings were conducted in PBS supplemented with 0.1% bovine serum albumin. Antibody validation and optimal antibody concentrations for stainings were pre-determined via titration.

Antibodies

List of antibodies used in this study is as follows:

Human/Mouse RASSF2 (R&D Systems, #AF5639)

MST1 (Cell Signaling, #3682)

MST2 (Cell Signaling, #3952)

Phospho-MOB1 (Thr35) (Cell Signaling, #8699)

MOB1 (E1N9D) (Cell Signaling, #13730)

Phospho-LATS1 (Thr1079) (Cell Signaling, #8654)

Phospho-LATS1 (Ser909) (Cell Signaling, #9157)

LATS1 (C66B5) (Cell Signaling, #3477)

DOCK2 (E-7) (Santa Cruz, #sc-365242)

Rac1/2/3 (Cell Signaling, #2465)

Phospho-p44/42 (ERK1/2) (Thr202/Tyr204) (D13.14.4E) (Cell Signaling, #4370)

p44/42 MAPK (ERK1/2) (Cell Signaling, #9102)

Phospho-AKT (Ser473) (Cell Signaling, #4060)

AKT (Cell Signaling, #9272)

Monoclonal Anti-Flag M2 (Sigma, #F3165)

Monoclonal Anti-HA (HA-7) (Sigma, #H9658)

β-Actin clone AC-15 (Millipore Sigma, #A1978)

Alpha-Tubulin (DSHB, #12G10)

Lamin B1 (B-10) (Santa Cruz, #sc-374015)

IRDye 800CW anti-mouse IgG (Li-Cor)

IRDye 800CW anti-rabbit IgG (Li-Cor)

IRDye 800CW anti-goat IgG (Li-Cor)

IRDye 680LT anti-mouse IgG (Li-Cor)

IRDye 680LT anti-rabbit IgG (Li-Cor)

IRDye 680LT anti-goat IgG (Li-Cor).

Statistical Analysis

Statistical analyses were conducted using GraphPad Prism (V 7.0) software. Individual statistical tests used for data analysis are indicated in figure legends. All data are displayed as mean (bar graph, horizontal line, or point) with error bars always representing S.E.M. All student's t-tests are conducted as two-tailed tests. Statistical significance in figures is displayed as follows: * = $p < 0.05$, ** = $p < 0.01$, *** = $p < 0.001$. In some cases, where p values are close to reaching a statistical significance threshold, exact values are displayed in figure panels. Where applicable, mouse sample sizes for measurement of various blood parameters were determined based on a minimal meaningful effect size of one standard deviation from a distribution of healthy wild-type control mice, assuming power = 0.9 and $\alpha = 0.05$.

Supplementary Figure Legends

Supplementary Fig. 1: Expression and transcriptional regulation of *RASSF2* in normal human hematopoiesis

a, *RASSF2* transcript expression across healthy primary human tissue, ranked by abundance. Displayed data are from the human genotype-tissue expression (GTEX) portal[65]. TPM, transcripts per million.

b, ChIP-seq tracks showing binding of indicated transcription factors within the *RASSF2* genomic locus in normal human CD34+ cells. ChIP-seq peaks are derived from [27].

Supplementary Fig. 2: Characterizing the re-expression of RASSF2 in models of t(8;21) AML

a, Western blot for indicated proteins. For SKNO-1 and Kasumi-1 cell lines, cells are transduced with retroviral expression vectors, MSCV-IRES-Puro^R (Vector) or MSCV-RASSF2-IRES- Puro^R (RASSF2), followed by selection with puromycin for three days.

b, Experimental schematic of the colony formation / serial replating in vitro assay of RUNX1-ETO mediated leukemic transformation.

c,d, Peripheral GFP+ cell frequency of indicated mice from experiment described in **(Fig. 3d)** at **(c)** 28 days post-transplantation, and **(d)** 125 days post-transplantation, solid lines indicate population mean.

e, Peripheral Hematocrit measurement of indicated mice from experiment described in **(Fig. 3d)** at 125 days post-transplantation, solid lines indicate population mean.

f, Representative Wright-Giemsa stained cytospins from moribund leukemic splenocytes of the indicated mice from experiment described in **(Fig. 3d)**.

Supplementary Fig. 3: RASSF2-mediated functions are unrelated to oncogenic Ras signaling or nucleo-cytoplasmic shuttling in AML

a, Western blots from cellular fractionation lysates for endogenous proteins in cell lines as indicated. Cyt, cytoplasmic fraction, Nuc, nuclear fraction. Data are representative of two experiments.

b, as **(a)**, but with stable transduction of MSCV-IRES- Puro^{R} (CTRL) or MSCV-Flag-RASSF2-IRES-Puro^{R} (RASSF2) in SKNO-1 cells.

c, as **(b)**, but with stable transfection of indicated vectors in HEK293T cells.

d, Western blots for indicated proteins and phospho-proteins in Kasumi-1 (left) and SKNO-1 (right) AML cell lines with stable transduction of MSCV-IRES- Puro^{R} (CTRL) or MSCV-Flag-RASSF2-IRES-Puro^{R} (RASSF2). Cells are serum starved overnight, and then stimulated with recombinant human IL-3 (20 ng/mL) and SCF (50 ng/mL) for times indicated. Data are representative of three experiments.

e, *RASSF2* mRNA expression from TCGA AML patient cohort, stratified based on presence or absence of activating mutation in *NRAS* (left) or *KRAS* (right). Data are visualized from cBioPortal (www.cbioportal.org).

Supplementary Fig. 4: RASSF2 does not mediate canonical Hippo pathway signaling

a, Western blots for indicated proteins and phospho-proteins in Kasumi-1 and SKNO-1 AML cell lines with stable transduction of MSCV-IRES- Puro^{R} (CTRL), MSCV-Flag-RASSF2-IRES-Puro^{R} (RASSF2), or MSCV-Flag-RASSF2ΔSARAH-IRES-Puro^{R} (RASSF2ΔSARAH). Data are representative of three experiments.

b, Quantification of multiple experiments described in **(a)**. Signal intensities are normalized to β-Actin loading and plotted relative to MSCV-IRES- Puro^{R} (CTRL) cells, which are indicated with dashed gray lines.

Supplementary Fig. 5: Mapping the RASSF2-proximal proteome by proximity-based biotin labeling (BioID2)

a, Schematic for RASSF2-BioID2 fusion protein inserts tested for use in proximity-based biotin labeling assay. All three inserts were cloned into MSCV-based retroviral expression vectors for expression of AML cell lines.

b, Western blot showing indicated protein amounts following stable transduction of Kasumi-1 AML cell line with vectors indicated in **(a)**. Data are representative of two experiments.

c, Western blot showing detection of RASSF2-13xL-BioID2 fusion protein and total biotinylated proteins (probed with Streptavidin) following transfection in HEK293T cells and supplementation with 50 μM biotin in the culture media for 24 hours. Data are representative of two experiments.

d, Western blot showing detection of total biotinylated proteins (probed with Streptavidin) following stable transduction in the Kasumi-1 cell line and supplementation with 50 μM biotin in the culture media for 48 hours. The three independent replicates that were submitted for mass spectometry are included. Biotinylated RASSF2-BioID2 fusion and MST1/2 bands are indicated.

a

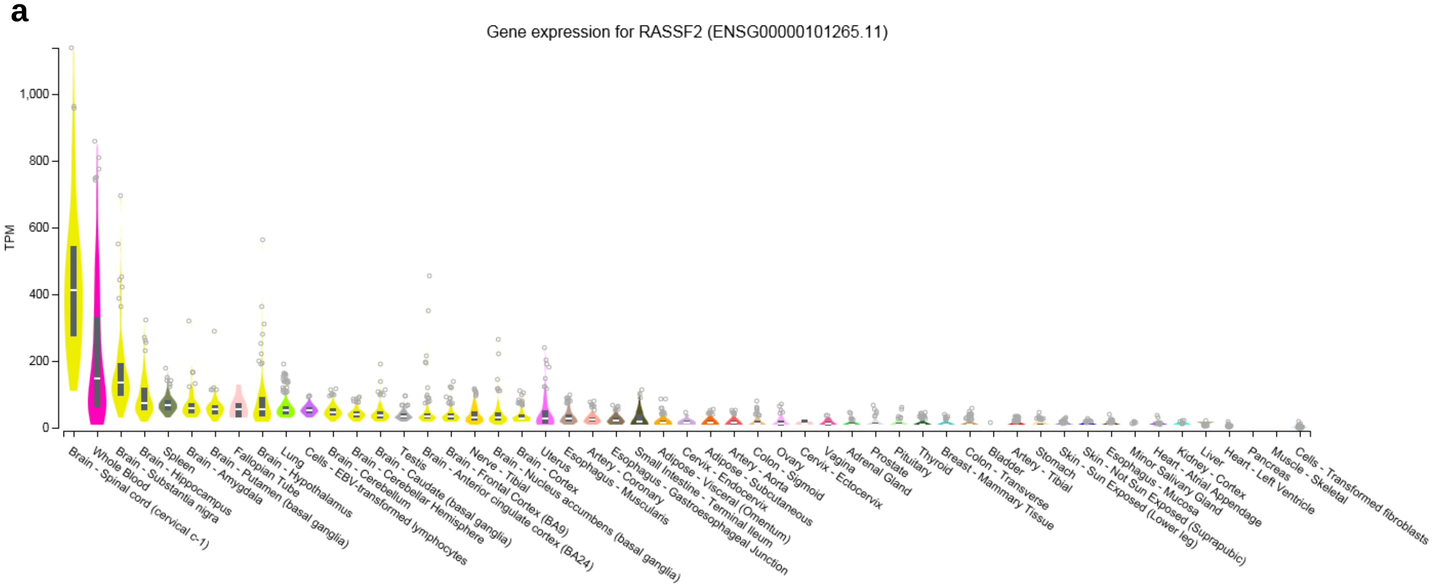

b

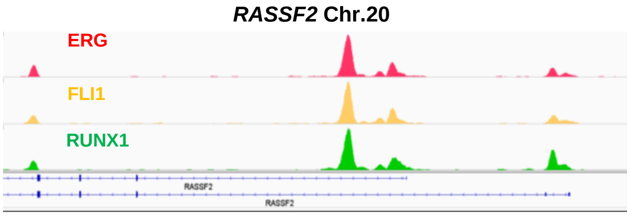

SUPPLEMENTARY FIGURE 1

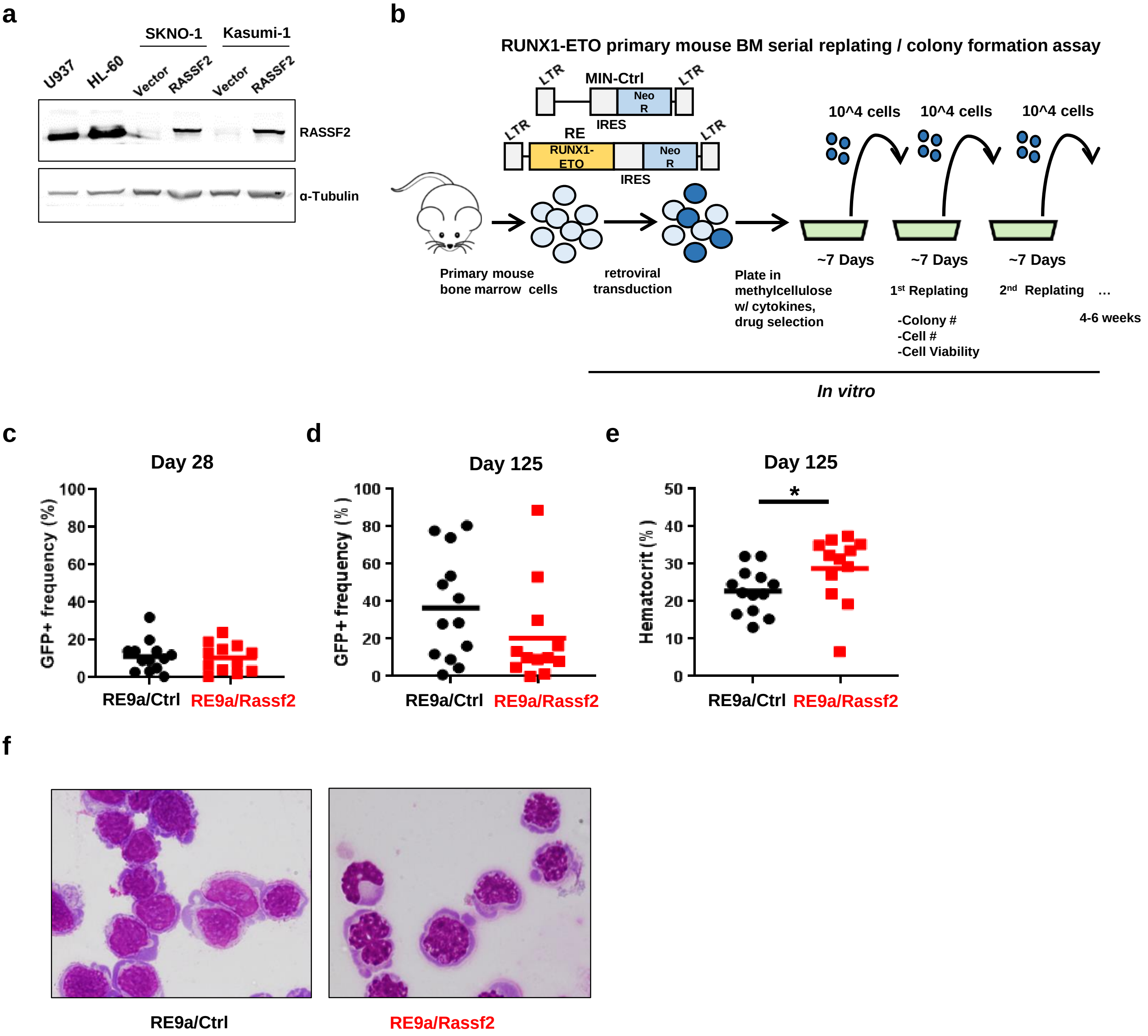

SUPPLEMENTARY FIGURE 2

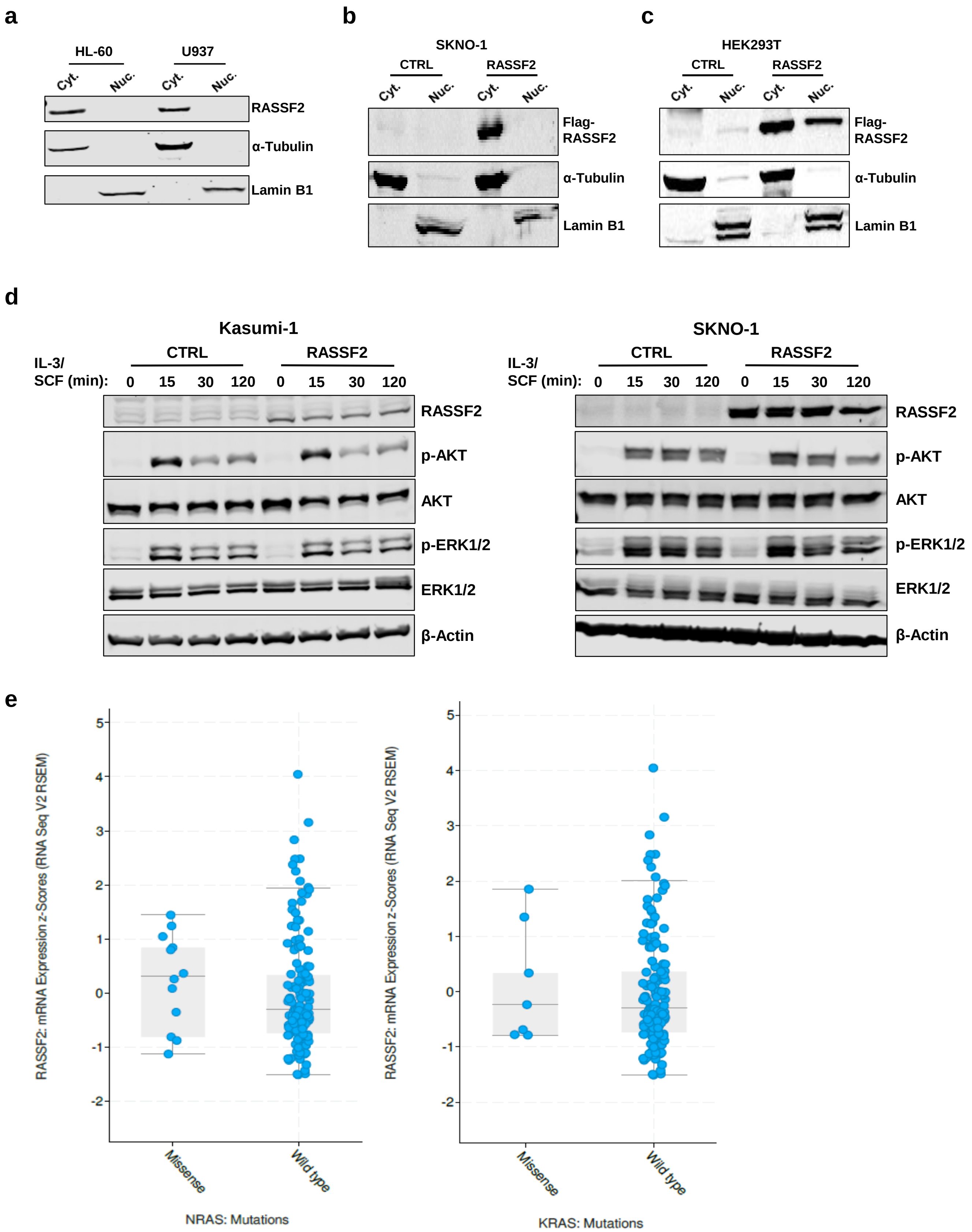

SUPPLEMENTARY FIGURE 3

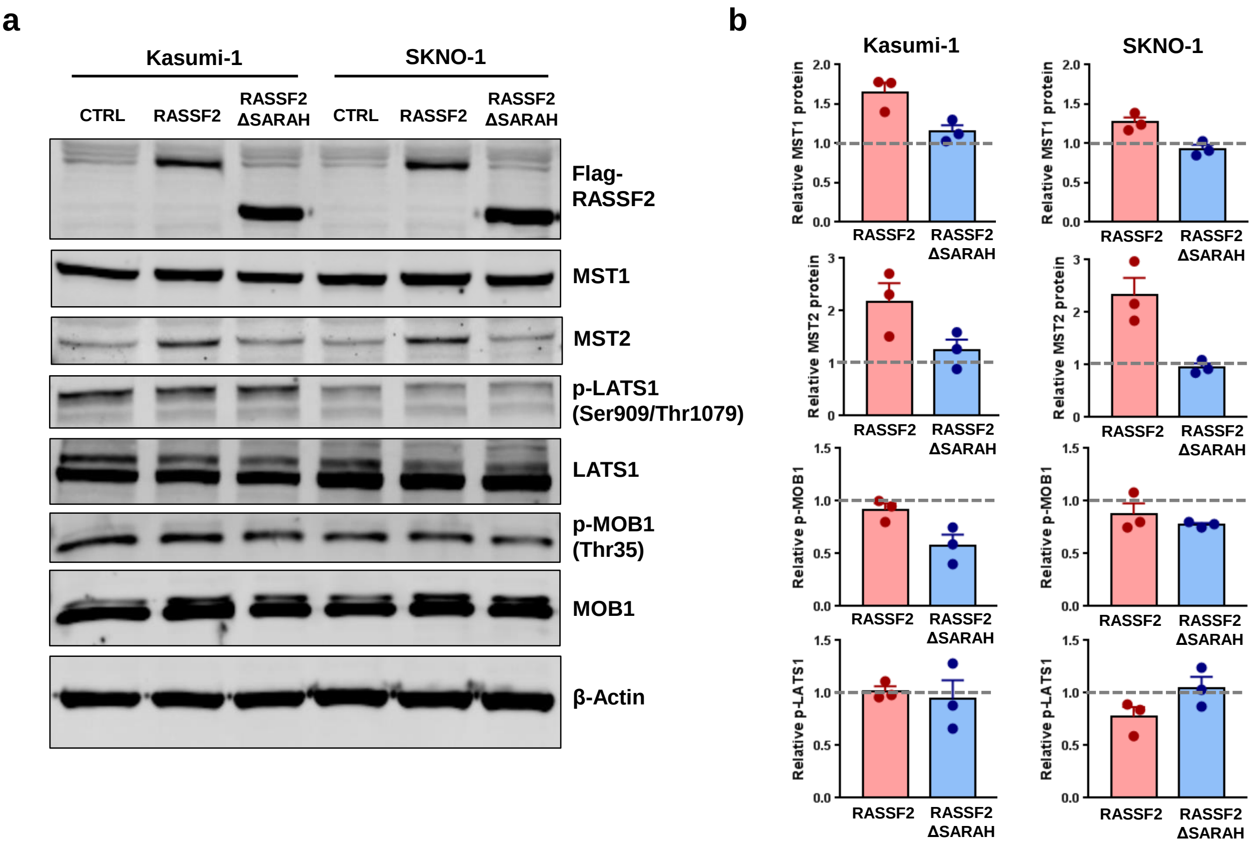

SUPPLEMENTARY FIGURE 4

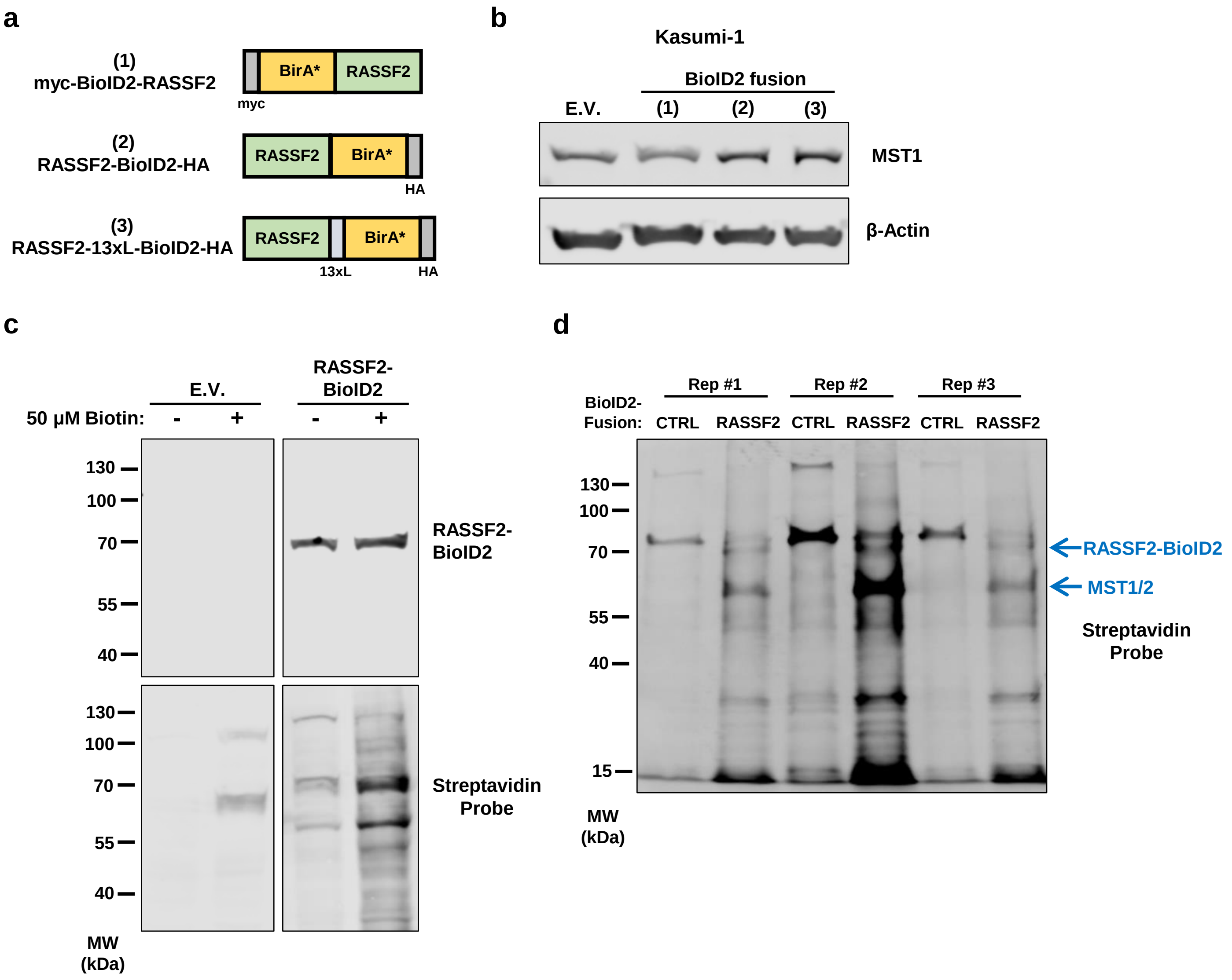

SUPPLEMENTARY FIGURE 5
